# Single‐Cell Immune Profiling Suggests Non‐Classical Monocyte Is Associated Immunodepression in Tuberculosis Treatment Non‐Responders

**DOI:** 10.1111/jcmm.71345

**Published:** 2026-08-31

**Authors:** Yiqun Xiong, Yahong Qu, Ying Wang, Dongliang Zhang, Hao Shen, Weixin Wang, Junshun Gao, ZhenBo Wang, Zhihong Shen, Yang Che

**Affiliations:** ^1^ Department of Infectious Diseases Ningbo Hospital of Integrated Traditional Chinese and Western Medicine Ningbo Zhejiang China; ^2^ Institute of Tuberculosis Prevention and Control, Ningbo Municipal Center for Disease Control and Prevention Ningbo Zhejiang China; ^3^ Cosmos Wisdom Biotech Co. Ltd Hangzhou China

**Keywords:** GALECTIN, IL‐16, immunosuppression, non‐classical monocytes, single‐cell RNA sequencing, sputum culture conversion, TRAIL, tuberculosis

## Abstract

Sputum culture conversion (SCC) at 2 months is an early indicator of tuberculosis (TB) treatment response, yet the associated immune alterations remain incompletely defined. We performed single‐cell RNA sequencing on peripheral blood mononuclear cells from eight TB patients, stratified by 2‐month SCC status. Non‐responders showed a relative enrichment of non‐classical monocytes and higher TB progression risk scores. CellChat analysis inferred altered IL16 and TRAIL communication involving these cells. CD4+ Tregs in non‐responders showed higher LGALS9 expression and inferred GALECTIN signalling towards cytotoxic lymphocyte subsets; cell‐level LGALS9 co‐expression correlated with HAVCR2 and TIGIT in non‐responders. Mature NK subclusters showed distinct enrichment profiles. These results describe an observational, transcriptomic and computationally inferred immune network associated with early treatment non‐response. They generate candidate biomarkers and hypotheses for prospective protein‐level and functional validation.

## 1 Background

The prevalence of Tuberculosis (TB) and its unfavourable treatment outcome posed a threat to the World Health Organization (WHO) End TB Strategy targets by 2035 [1].

Unfavourable treatment outcomes are influenced by multiple factors, including bacteriological characteristics, demographics, clinical history, health care delivery and socioeconomic conditions [2, 3]. Patients with drug‐resistant TB (DR‐TB) experience a higher proportion of poor outcomes than those with drug‐sensitive TB [4, 5]. Individual risk factors—such as sex, age, education, prior TB treatment, substance use and resistance to fluoroquinolones—also influence sputum conversion rates (SCR) and the likelihood of achieving sputum culture conversion (SCC) [6, 7].

SCC, defined as two consecutive negative cultures collected at least 30 days apart [8], is a critical early indicator of treatment efficacy and a predictor of long‐term cure [9, 10]. Monitoring SCC allows earlier identification of treatment failure and delayed conversion increases the risk of transmission within communities, highlighting its public health relevance [11, 12]. Despite its clinical importance, the immunological mechanisms that govern SCC remain poorly understood. Host–pathogen interactions are complex and heterogeneous, with significant interpatient variation reflected in radiographic presentation, symptoms and disease outcomes [13, 14]. It is unclear how these immune interactions and molecular pathways influence the timing or likelihood of sputum conversion.

Single‐cell RNA sequencing (scRNA‐seq) offers a high‐resolution approach to investigate immune heterogeneity and cellular responses at the single‐cell level [15]. Previous transcriptional profiling in TB has revealed differences in the state and abundance of immune cell subpopulations early in disease [16], and scRNA‐seq has identified functional variation across affected tissues that may act as molecular switches influencing disease progression [17]. However, few prospective studies have systematically linked SCC status with single‐cell immune profiling to elucidate the underlying immune alterations.

Considering the crucial role of intercellular communication mediated by cytokines, chemokines and other signalling molecules in TB pathogenesis and treatment response, we applied scRNA‐seq to peripheral blood mononuclear cells (PBMCs) from primary TB patients stratified by 2‐month SCC results. This approach aimed to comprehensively characterize the cellular and molecular heterogeneity associated with early treatment response, potentially revealing immunological determinants of SCC.

## 2 Methods

### 2.1 Sample Collection and Processing

All enrolled patients were confirmed to have drug‐sensitive *Mycobacterium tuberculosis* infection based on drug susceptibility testing. None of the patients had drug‐resistant TB.

### 2.2 PBMC Isolation

The scRNA‐seq experiment was conducted at the laboratory of Cosmos Wisdom Biotech Co. Ltd. PBMCs were isolated from peripheral blood using LeucoSep tubes prefilled with Ficoll‐Paque Plus. Samples were centrifuged at 700 g for 20 min, and PBMCs were collected from the interface, followed by red blood cell lysis and two washes in PBS with 0.04% BSA. Cell suspensions were filtered through 30‐μm strainers, and only cells with > 90% viability were used for downstream processing.

### 2.3 Sample Fixation and Hybridization

PBMCs were fixed for 24 h at 4°C and quenched. For each sample, 1 × 10^6^ fixed cells were incubated with unique Human WTA Probes for 24 h, washed, counted and pooled to achieve equal representation. Samples were then processed immediately for GEM generation.

### 2.4 Single‐Cell Sequencing

Fixed RNA libraries were prepared using the 10 × Genomics Chromium X Instrument and Chromium Fixed RNA Kit. Hybridized samples were partitioned into Gel Beads‐in‐emulsion (GEMs), barcoded and ligated probes were pre‐amplified. Libraries were quantified with Qubit and sized using Qsep100. Sequencing was performed on an Illumina NovaSeq X Plus (2 × 150 bp).

### 2.5 Single‐Cell RNA Statistical Analysis

Raw reads were filtered with fastp, aligned to the human probe set (GRCh38‐2020‐A) using CellRanger multi v7.1.0 and aggregated across samples. Cells with > 200 expressed genes and < 20% mitochondrial reads passed quality filtering. Mitochondrial genes were then excluded from the downstream expression matrix; mitochondrial percentage was not regressed during normalization or scaling. Doublets were identified and excluded using Scrublet with a pre‐specified conservative score threshold of 0.25 applied identically to all samples [18]; no group‐specific thresholding was performed. Genes expressed in fewer than three cells were filtered out.

Normalization and scaling were performed using Scanpy (v1.9.3). No mitochondrial‐percentage regression was applied after mitochondrial genes were excluded. PCA (top 30 PCs) and UMAP were used for dimensionality reduction and visualization. Unsupervised clustering identified cell populations, and marker genes were detected using the Wilcoxon rank‐sum test (Log2FC > 0.25, *p* < 0.05, pct_nz_group > 0.1). Clusters of the same cell type were reanalyzed for refined annotation.

### 2.6 Functional and Pathway Analysis

GSEApy package (version: 1.1.1, https://github.com/zqfang/GSEApy/releases) was used for gene set enrichment analysis [19]. We utilized the scanpy.tl.score_genes function from the Scanpy package to compute feature scores for predefined gene sets [20, 20]. To assess the tissue preference of each cell subset, we calculated the ratio of observed to expected cell numbers (Ro/e) [21].

### 2.7 SCENIC Analysis

To assess transcription factor regulation strength, we applied the Single‐cell regulatory network inference and clustering (pySCENIC, v0.9.5) workflow, using the 20‐thousand motifs database for RcisTarget and GRNboost [22].

### 2.8 Trajectory and Differentiation Analysis

Single‐cell trajectories were reconstructed using Monocle2 (DDR‐Tree) [23], CytoTRACE [24] and Scanpy‐based PAGA. Marker genes from Scanpy clustering and normalized expression matrices were used as input. CytoTRACE scores estimated differentiation potential, and PAGA connectivity inferred developmental transitions, validated by lineage‐specific marker expression.

### 2.9 CellChat Cell–Cell Communication Analysis

Intercellular signalling was inferred using CellChat (v1.6.1) based on known ligand‐receptor pairs from CellChatDB.human [25]. Outgoing and incoming signalling changes were compared between groups. CellChat results were interpreted as transcript‐informed predictions of potential ligand‐receptor communication; they do not quantify protein abundance, establish signal directionality independent of cell abundance or prove functional causality.

### 2.10 Microarray Data Analysis

Microarray dataset GSE40553 was used as exploratory longitudinal treatment‐time context. CIBERSORT estimated immune‐cell subset abundance (perm = 100, quantile normalization disabled); samples were clustered by Euclidean distance, and expression differences were assessed with the Wilcoxon rank‐sum test. CIBERSORT estimates cannot precisely resolve non‐classical monocytes from all related myeloid subsets, and the bulk‐transcriptome analysis was not considered validation of single‐cell‐defined functional states. No individual‐level 2‐month SCC outcome was used in this external analysis.

Patient‐level visualization and standardized effects: supplied sample‐level composition and expression panels are presented for four non‐responders and four responders. Cohen's d for major immune‐cell proportions was defined as the non‐responder minus responder mean divided by the pooled sample standard deviation; positive values indicate higher mean proportions in non‐responders. With *n* = 4 per group, effect sizes are descriptive and do not replace patient‐level validation.

Exploratory virtual knockout analysis: scTenifoldKnk was applied separately to IL16 and TNFSF10 in the inferred transcriptomic network. Supplied results with adjusted *p* < 0.05 were summarized as network perturbations. This in silico analysis is hypothesis‐generating and does not replace experimental perturbation, protein validation or assessment of clinical feasibility and safety.

## 3 Results

### 3.1 Single‐Cell Transcriptomic Profiles of Immune Cells in Response to Standard TB Treatment

**FIGURE 1:**
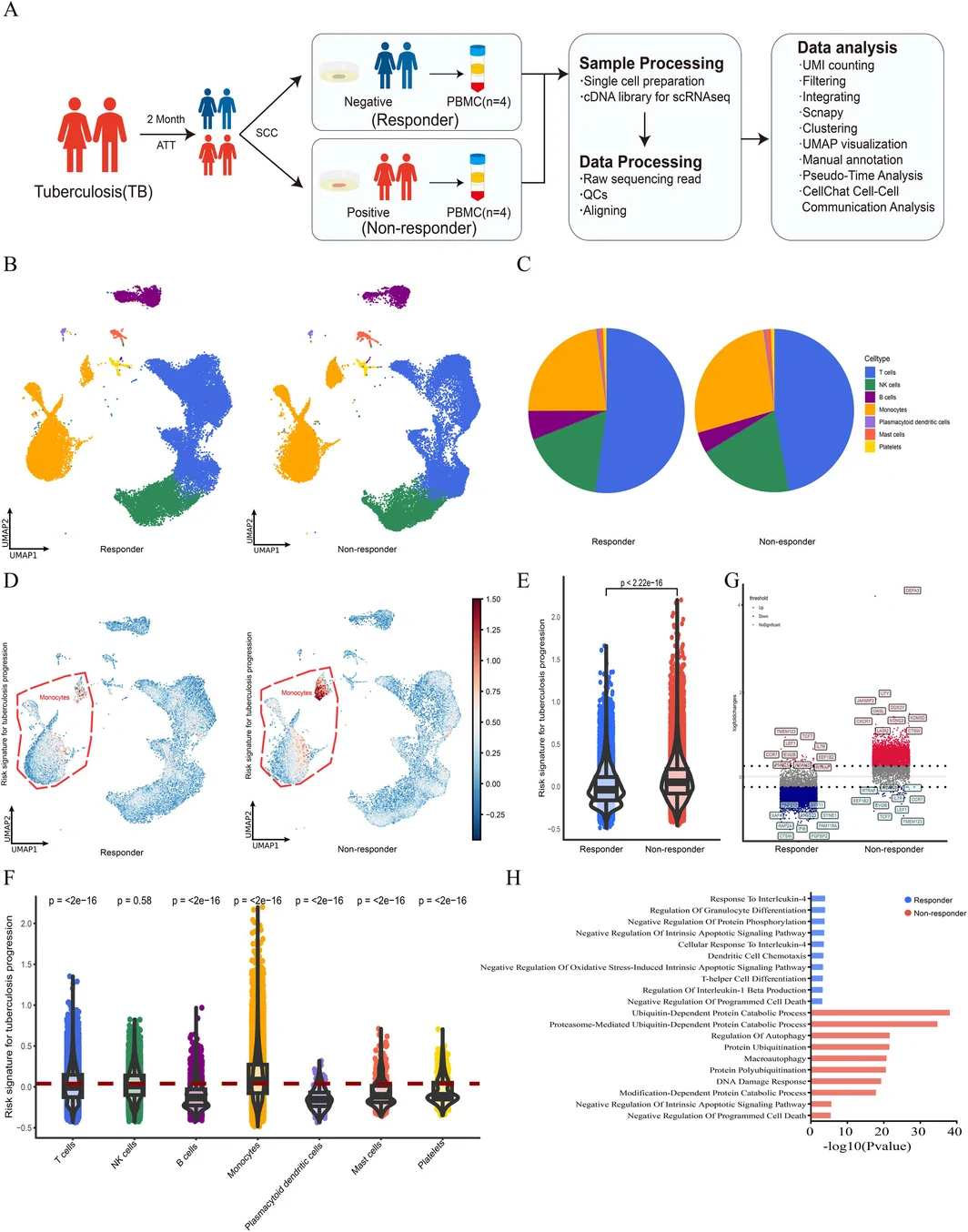
Single‐cell transcriptomic landscape of PBMCs from TB treatment responders and non‐responders. (A) Schematic overview of the study design. Eight TB patients were recruited and classified into treatment responders (Control, *n* = 4) and non‐responders (non‐responders, *n* = 4) based on sputum culture conversion (SCC) status at 2 months after anti‐tuberculosis therapy (ATT). (B) UMAP plot of 83,999 PBMCs revealing 17 transcriptional clusters. Cells were annotated into six major immune cell types based on canonical marker genes: T cells, NK cells, B cells, myeloid cells, mast cells and platelets. (C) Proportions of each major immune cell type in the Control and non‐responders groups. (D) Comparison of TB progression risk scores between Control and non‐responders groups. (E) Violin plots of individual patient risk scores. Statistical significance was assessed using the Wilcoxon rank‐sum test. (F) Violin‐box‐scatter plots showing the distribution of TB risk scores across immune cell types. Each point represents a single cell. The red horizontal line indicates the overall mean risk score across all PBMCs. Statistical comparisons were performed between each cell type and the total PBMC population using the Wilcoxon rank‐sum test. (G) Differential gene expression analysis between Control and non‐responders groups across all PBMCs. The top 10 most significantly upregulated genes in each group are labelled. (H) Pathway enrichment analysis showing distinct biological processes activated in the Control and non‐responders groups.

We recruited 8 TB patients, including treatment responders (*n* = 4) and non‐responders (*n* = 4), stratified by 2‐month SCC status (Figure 1A). Clinical characteristics including age, sex, treatment history and drug susceptibility were collected (Table S1; Figure S1A). After quality control, 83,999 PBMCs were retained (40,918 responders; 42,981 non‐responders), with an average of 1723 genes detected per cell. PCA and UMAP with Leiden clustering identified 17 clusters, which were annotated into six major immune cell types: T cells, NK cells, B cells, myeloid cells, mast cells and platelets (Figures 1B and S1B; Table S2). Cell composition analysis indicated higher proportions of monocytes, NK cells and mast cells in the non‐responders group, although differences were not statistically significant (Figures 1B,C and S1C; Table S2).

To evaluate inter‐individual variability, we quantified non‐classical monocyte (NCM) composition and IL16/TNFSF10 transcript expression for each biological replicate (four non‐responders and four responders; Figure S6A,B). The total monocyte fraction showed a large standardized between‐group difference (Cohen's d = 1.04; Figure S6C).

To evaluate disease severity, we applied a previously established TB progression risk signature (Table S3) [26]. The non‐responders group exhibited significantly higher risk scores, primarily driven by monocytes (Figure 1D–F). Expression of GBP2, STAT1 and TAP1 was elevated in monocytes from the non‐responders group (Figure S1D). Differential gene expression analysis revealed enrichment of T cell–associated genes (TCF7, CCR7) in the Control group, whereas DEFA3 and CXCR1 were upregulated in monocytes, and JAKMP2 and UTY in NK cells in the non‐responders group (Figure 1G; Table S2). Pathway analysis indicated activation of cell survival, anti‐apoptosis, protein degradation and NF‐κB signalling in the non‐responders group, while the Control group showed enrichment in immune regulation, cell differentiation and anti‐inflammatory or antioxidant pathways (Figure 1H). Gene set scoring further confirmed enhanced activation of antigen processing, immune response and innate and adaptive immune pathways in the non‐responders group, whereas antifungal and antiviral response pathways remained unchanged (Figure S1E).

### 3.2 Non‐Classical Monocytes Are Enriched and Terminally Differentiated in Treatment Non‐Responders

**FIGURE 2:**
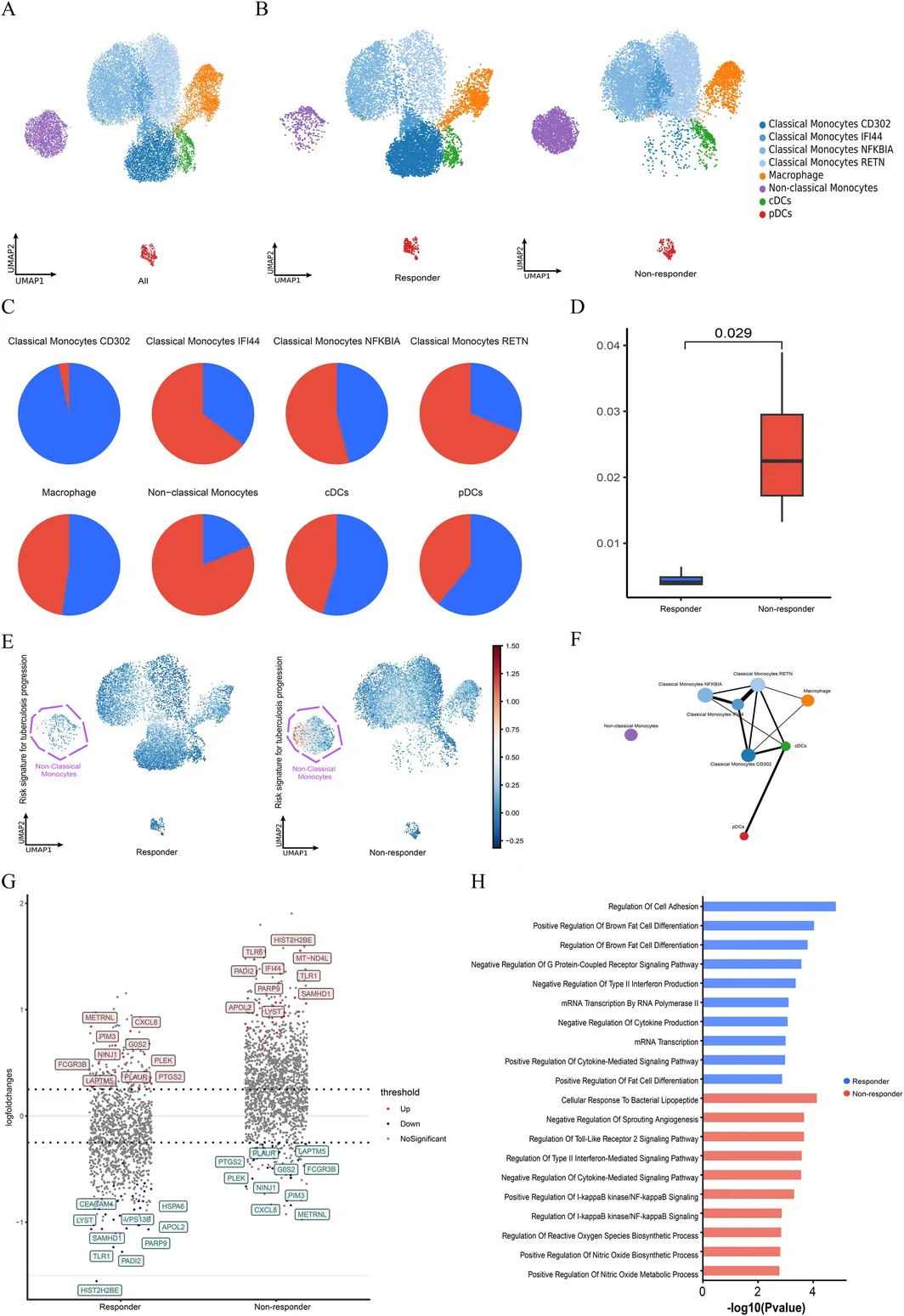
Non‐classical monocytes are enriched and terminally differentiated in treatment non‐responders. (A) UMAP plot showing the distribution of myeloid cells within the total PBMC population. (B) Proportions of each myeloid subset across individual samples in Control and non‐responders groups. (C) Pie charts comparing the relative proportions of myeloid subsets between Control and non‐responders groups. (D) Box plots showing the relative abundance of non‐classical monocytes between Control and non‐responders groups. (E) UMAP plots of TB progression risk scores in monocytes, stratified by group. (F) Trajectory inference analysis of myeloid subsets using PAGA. (G) Differentially expressed genes between non‐classical monocytes from Control and non‐responders groups. (H) Gene Ontology (GO) enrichment analysis of biological processes (BP) for differentially expressed genes in non‐classical monocytes.

Myeloid cells exhibited the most prominent transcriptional changes and risk score enrichment in the non‐responders group, prompting subcluster analysis that identified eight subsets (Figures 2A and S2A). Among these, non‐classical monocytes (NCMs) were relatively enriched in the non‐responders group, with elevated TB progression risk scores (Figures 2D,E and Figure S2B,C; Table S3). PAGA and Monocle3 trajectory analyses revealed no clear differentiation path, and CytoTRACE indicated that NCMs were terminally differentiated (Figures 2F and S2D,E). SCENIC analysis highlighted activation of key transcription factors, including ZNF177, FOXP3 and HES5 (Figure S2F).

The patient‐level NCM composition plot displays each biological replicate and permits assessment of between‐group heterogeneity without relying solely on cell‐level observations (Figure S6A).

Differential gene expression analysis showed upregulation of TLR6, TLR1, IFI44 and PARP9 in non‐responders' NCMs, whereas LAPTM5, CXCL8 and PTGS2 were higher in Control (Figure 2G). Gene ontology enrichment revealed that Control NCMs were associated with cell adhesion and positive regulation of cytokine‐mediated signalling, while non‐responders' NCMs were enriched for regulation of Toll‐like receptor 2 signalling, type II interferon‐mediated signalling, negative regulation of cytokine signalling and I‐κB kinase/NF‐κB pathways (Figure 2H). These findings suggest that NCMs in non‐responders are terminally differentiated and exhibit a transcriptional program biased towards innate immune activation and inflammatory signalling.

### 3.3 Reduced Outgoing and Enhanced Incoming Signalling of Non‐Classical Monocytes via IL16/TRAIL Pathways

**FIGURE 3:**
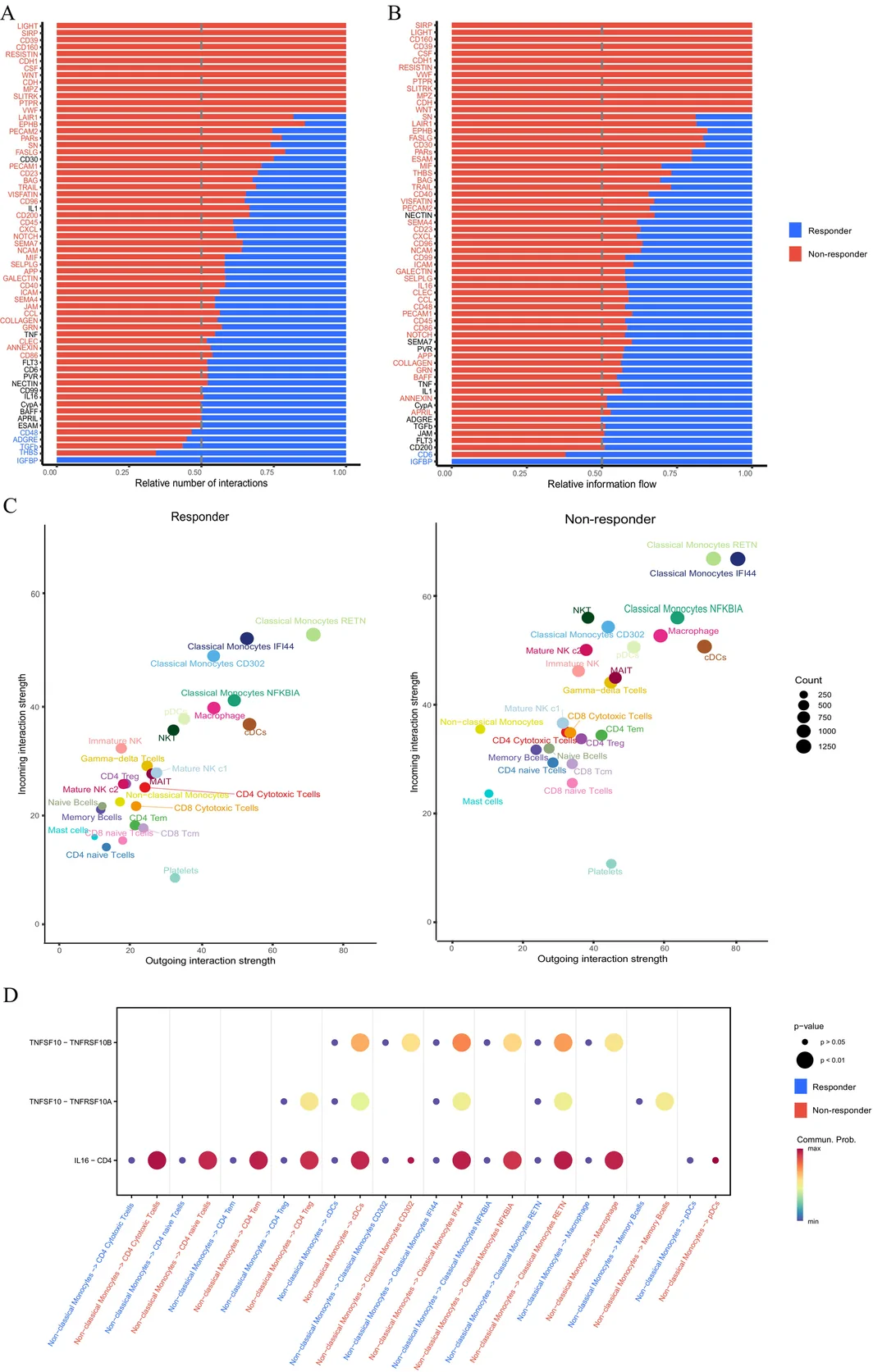
Altered signalling dynamics of non‐classical monocytes in non‐responders vs. Control groups. (A) Number of ligand‐receptor interaction pairs identified in non‐responders and Control groups. Black indicates no significance; red indicates non‐responders enrichment; blue indicates Control enrichment. (B) Weight of ligand‐receptor interaction pairs identified in non‐responders and Control groups. Black indicates no significance; red indicates non‐responders enrichment; blue indicates Control enrichment. (C) Comparison of incoming and outgoing signalling number and strength involving non‐classical monocytes as receivers and senders, respectively. (D) Dot plot showing enhanced IL16 and TRAIL signalling pathways with non‐classical monocytes acting as ligand sources.

We next examined CellChat‐inferred intercellular communication involving NCMs. Globally, non‐responders showed more inferred interactions and stronger overall communication than responders (Figure S3A). When NCMs were considered as ligand‐sending cells, the number and strength of inferred outgoing interactions were reduced in non‐responders, whereas inferred incoming signals from other cell types were stronger (Figure S3B,C). Comparative CellChat analysis identified IL16 and TRAIL (TNFSF10) pathways among upregulated inferred interactions involving non‐responder NCMs and classical monocyte subsets or CD4+ T cells (Figure 3A–D) [27, 28]. These results are transcriptomic ligand‐receptor hypotheses and do not demonstrate ligand secretion, cell‐autonomous source specificity or causal signalling.

### 3.4 Cytotoxic T Cell Expansion and LGALS9 ^+^ Treg‐Mediated Immunoregulation in Treatment Non‐Responders

**FIGURE 4:**
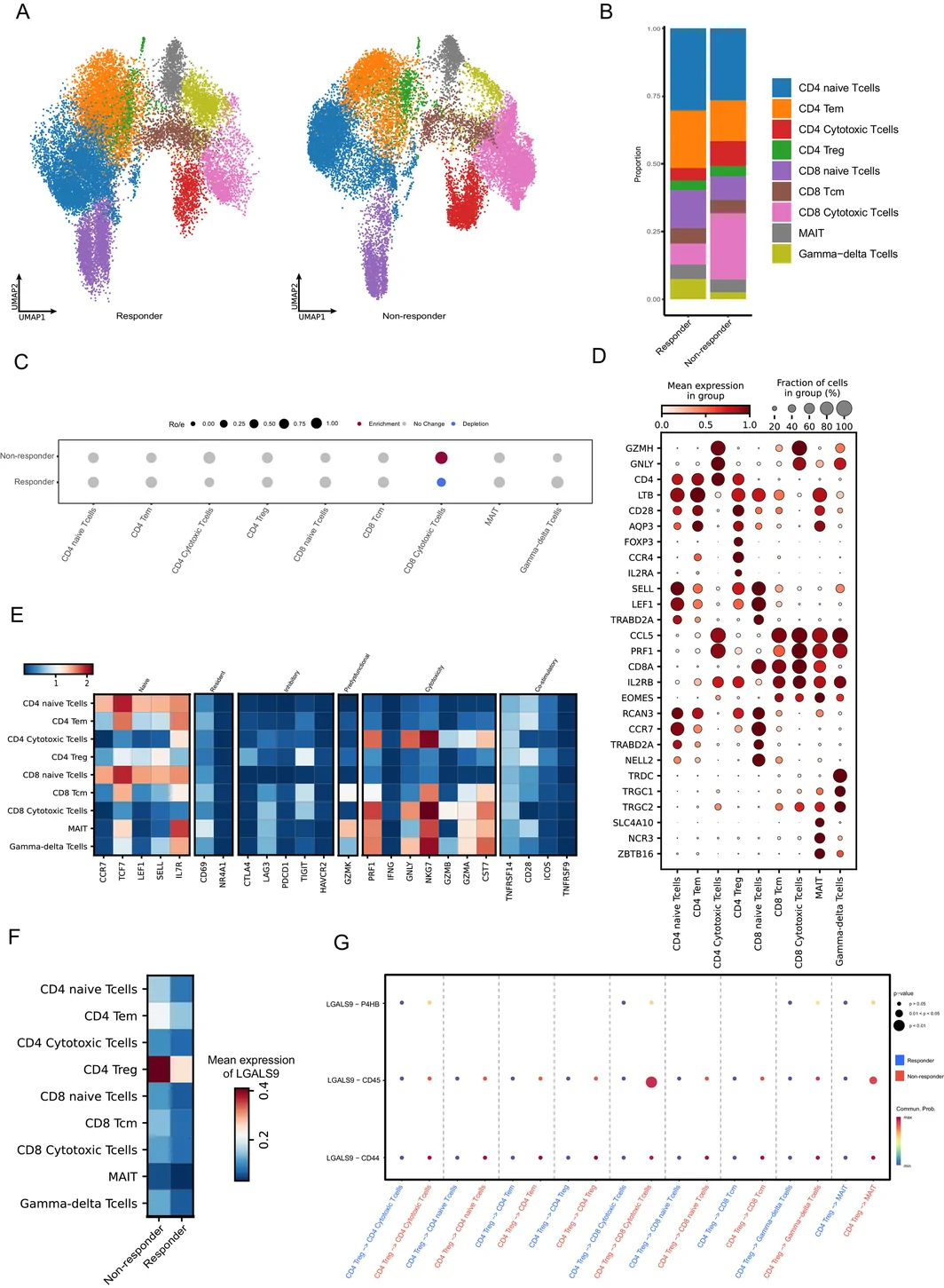
Cytotoxic T cell expansion and GALECTIN signalling‐mediated Treg immunoregulation in treatment non‐responders. (A, D) Subclustering and annotation of T cell subsets based on canonical markers. (B) Proportion plot showing the distribution of T cell subsets in each group. (C) Dot plot of Ro/e enrichment analysis, with enrichment defined as Ro/e > 1.5 and *p* < 0.05, depletion as Ro/e < 0.5 and *p* < 0.05 and no change otherwise. (E) Heatmap showing functional gene expression profiles across T cell subsets. (F) Heatmap displaying LGALS9 gene expression levels in each T cell subset by group. (G) CellChat analysis illustrating GALECTIN signalling from CD4^+^ Tregs (source) to other T cell subsets (receptors).

In addition to altered myeloid signalling, the T cell compartment showed notable changes. Overall T cell abundance was reduced in the non‐responders group, while subclustering revealed a significant increase in CD8^+^ cytotoxic T cells compared to Control (Figure 4A–D). Functional assessment indicated high cytotoxic potential in both CD8^+^ and CD4^+^ cytotoxic T cells, whereas CD4^+^ regulatory T cells (Tregs) exhibited stronger inhibitory profiles (Figure 4E; Table S4) [29]. To further characterize the exhaustion status of T cell subsets, we examined the expression of these inhibitory receptors based on a predefined set of exhaustion‐associated genes. UMAP visualization revealed distinct expression patterns: HAVCR2 was broadly expressed across multiple subsets with elevated levels in non‐responders (Figure S5A,B), while TIGIT showed more restricted expression, predominantly in CD8^+^ cytotoxic T cells and Tregs from the non‐responder group (Figure S5D,E). HAVCR2 expression increased in non‐responders, particularly in CD8^+^ cytotoxic T cells and CD4^+^ Tregs (Figure S5C); a similar trend was observed for TIGIT (Figure S5F). LGALS9 expression was significantly upregulated in non‐responders Tregs (log_2_FC = 0.85, p_adj = 6.41e‐06). CellChat analysis inferred that non‐responders Tregs mediated LGALS9‐dependent interactions—including LGALS9–P4HB, LGALS9–CD45, and LGALS9–CD44—primarily targeting CD8^+^ cytotoxic T cells, consistent with enhanced Treg‐associated immunoregulation in non‐responders (Figure 4G).

At the single‐cell level, LGALS9 expression was positively correlated with HAVCR2 in non‐responders (*R* = 0.471, *p* = 3.74e‐06), whereas the corresponding association in responders was weaker and non‐significant (*R* = 0.236, *p* = 0.244). LGALS9 was also positively correlated with TIGIT in non‐responders (*R* = 0.420, *p* = 4.46e‐05) but not in responders (*R* = 0.193, *p* = 0.343; Figure S7A,B).

### 3.5 Functional Exhaustion and Altered Crosstalk of NK Cell Subsets in Treatment Non‐Responders

**FIGURE 5:**
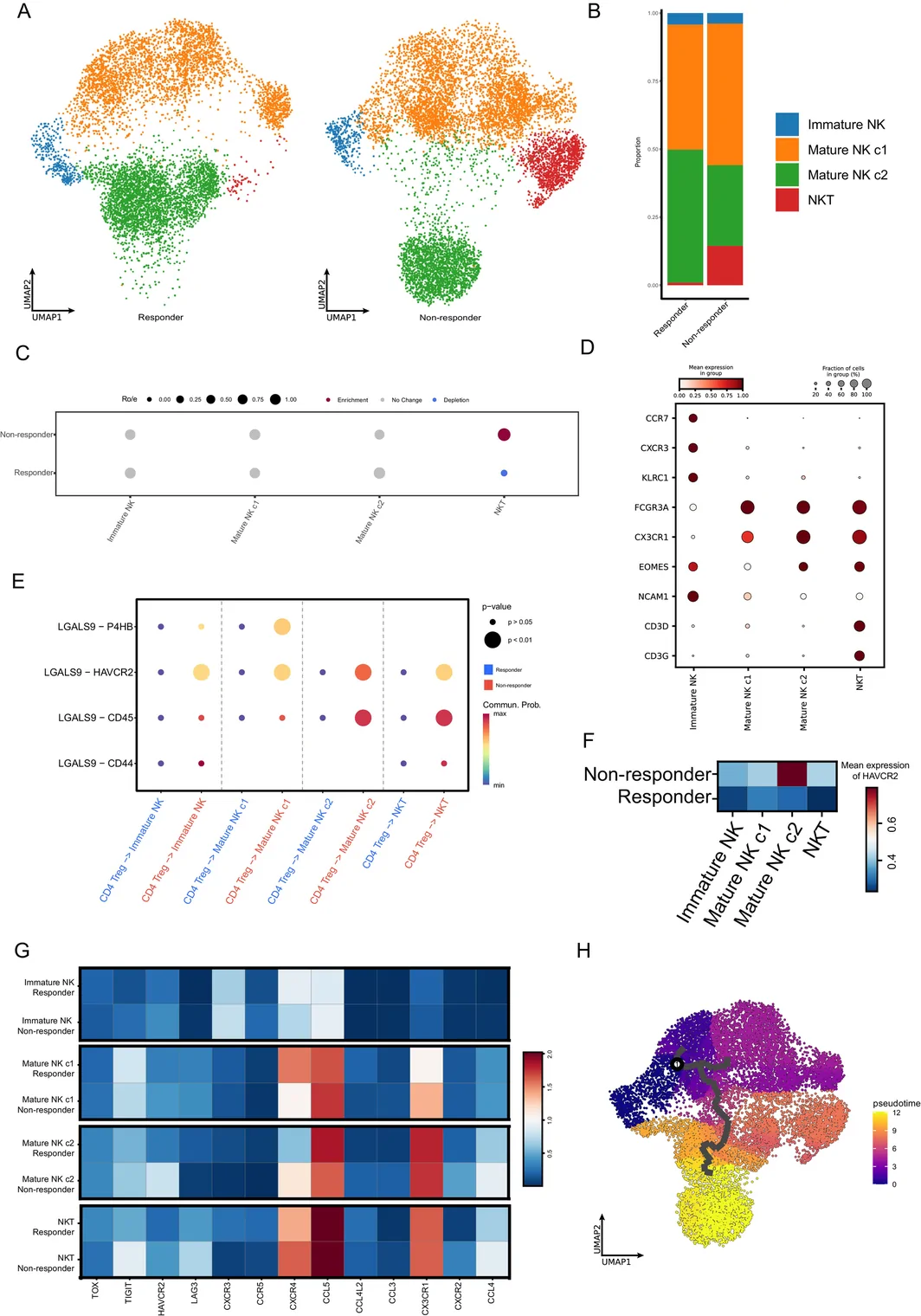
Altered innate lymphocyte composition and GALECTIN signalling in treatment non‐responders. (A, D) Subclustering and annotation of NK cell subsets into Immature NK and Mature NK populations based on canonical markers. (B) Proportion plot showing the distribution of NK cell subsets in each group. (C) Dot plot of Ro/e enrichment analysis, with enrichment defined as Ro/e > 1.5 and *p* < 0.05, depletion as Ro/e < 0.5 and *p* < 0.05 and no change otherwise. (E) CellChat analysis illustrating GALECTIN signalling from CD4^+^ Tregs (source) to NK cell subsets (receptors). (F) Gene expression levels of HAVCR2 in NK cell subsets across groups. (G) Heatmap showing upregulation of exhaustion markers in NK cell subsets across groups. (H) Monocle 3 pseudotime trajectory analysis visualized by UMAP plot.

We next examined innate lymphocytes, focusing on NK‐cell clusters classified as immature and mature NK subsets (Figure 5A,D). NKT cells were more abundant in non‐responders, and the UMAP showed spatial separation of Mature NK c2 between groups (Figure 5B,C). CellChat inferred stronger GALECTIN signalling, including LGALS9‐HAVCR2 interactions from CD4+ Tregs to Mature NK c2, in non‐responders (Figure 5E). HAVCR2 expression was higher in Mature NK c2, TIGIT was higher in NKT cells, and CCL5 and CXCR4 were reduced (Figure 5F,G). Pseudotime placed these subsets towards the terminal end of the inferred trajectory (Figure 5H).

To define transcriptional programs associated with the two Mature NK subclusters, we performed KEGG enrichment analysis of their subcluster‐specific gene sets. Mature NK c1 was enriched for PD‐1/PD‐L1 checkpoint, T‐cell receptor and Toll‐like receptor signalling, whereas Mature NK c2 was enriched for natural killer cell‐mediated cytotoxicity, cellular senescence and MAPK signalling (Figure S8A,B). These enrichment results annotate subcluster‐specific gene sets and do not constitute treatment‐group comparisons.

### 3.6 Longitudinal GSE40553 Analyses Contextualize Treatment‐Associated Non‐Classical‐Monocyte Changes but Do Not Test SCC Outcome

**FIGURE 6:**
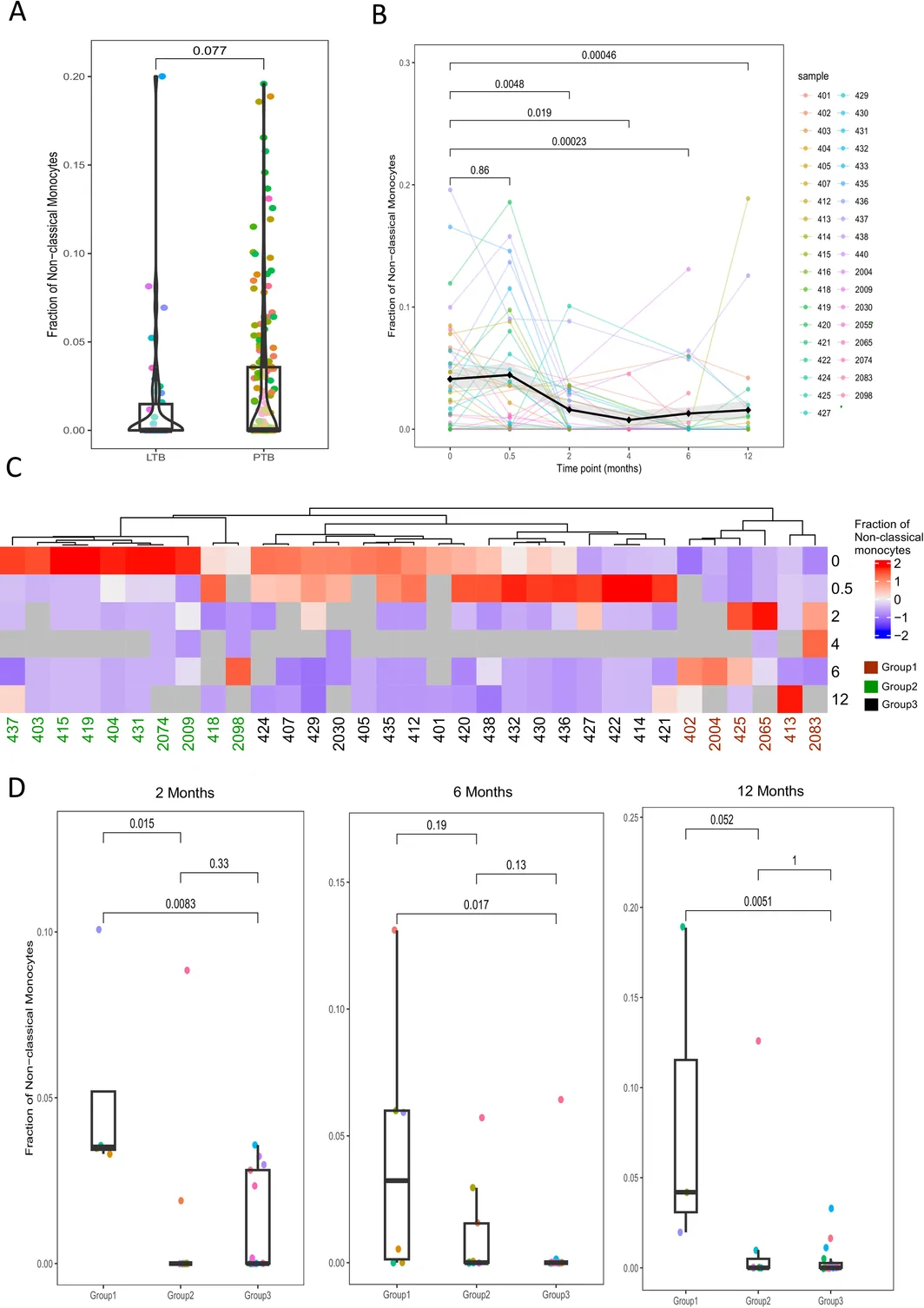
Longitudinal GSE40553 context for non‐classical‐monocyte estimates during anti‐TB treatment. (A) Comparison of estimated non‐classical‐monocyte proportions between latent tuberculosis (LTB) and pulmonary tuberculosis (PTB) samples. (B) Longitudinal changes in estimated non‐classical‐monocyte proportions during anti‐TB therapy in PTB samples. Each line represents an individual participant, and the black line denotes the overall mean with the shaded area indicating the standard error. This dataset is treatment‐time context only and was not analyzed as an individual‐level 2‐month SCC outcome‐validation cohort. (C) Hierarchical clustering of patients based on standardized non‐classical monocyte proportions across all time points. Distinct patient groups were identified, with different colours indicating the clustering results. (D) Comparison of non‐classical monocyte proportions at 2, 6 and 12 months across the identified clusters, with statistical testing performed using the Wilcoxon rank‐sum test.

We used GSE40553 as exploratory longitudinal treatment‐time context rather than as an SCC‐outcome validation cohort [30]. No individual‐level 2‐month SCC outcome was used in this analysis. CIBERSORT estimates showed no significant difference in non‐classical‐monocyte proportions between LTB and PTB (*p* = 0.077) or between baseline and 2 weeks (*p* = 0.86), followed by a decrease at 2 months (*p* = 0.0048) that remained low through 12 months (Figures 6A,B, S4A–D). Given the limits of bulk deconvolution for closely related myeloid subsets, these data provide descriptive temporal context only and cannot independently validate the non‐responder association or the single‐cell functional states.

### 3.7 Exploratory Virtual Knockout Analysis of IL16 and TNFSF10

To explore candidate network‐level consequences of perturbing the inferred ligands, we applied scTenifoldKnk virtual knockout analysis. IL16 and TNFSF10 virtual knockout yielded 24 and 34 FDR‐significant perturbation‐associated genes, respectively. Representative IL16‐associated genes included TCF7, TRBC2, LTB, TRAC, IL7R, ETS1, CCR7 and CD3E, whereas TNFSF10‐associated genes included NKG7, PRF1, CST7, IL2RB, GNLY, ADGRG1, ARL4C and TRBC2 (Figure S9A,B; Table S5). Ninety perturbation‐associated genes were shar ed. between the two analyses; 86 mapped genes were enriched for cellular defence response (GO:0006968; adjusted *p* = 1.24e‐07), regulation of peptidase activity (adjusted *p* = 1.24e‐07), and response to molecule of bacterial origin (adjusted *p* = 7.41e‐07; Figure S9C). These in silico results prioritize candidates for experimental study and do not establish gene function, protein effects, therapeutic benefit or safety.

## 4 Discussion

Single‐cell analysis revealed a striking expansion and phenotypic dysregulation of NCMs in TB patients who failed to achieve sputum culture conversion after 2 months of HREZ therapy (non‐responders) (Figures 1D–F, 2E, S2C). Consistent with prior reports linking non‐classical monocyte expansion to TB disease severity, we observed marked enrichment of this population in non‐responders [31]. The TB progression risk signature score shows that NCMs have been previously associated with advanced disease and impaired bacterial clearance in active TB (Figures 2C,D and S2B) [26]. In line with findings by Ong and Hadadi et al., which demonstrated senescence‐like features of NCMs driven by NF‐κB and IL‐1α signalling, our data corroborate these observations (Figure 1H) [32]. GBP2, STAT1, and TAP1 have previously been proposed as potential biomarkers for MTB infection [33, 34, 35]. In our study, we found that the expression levels of these genes in NCMs were higher in treatment non‐responders compared with the corresponding responders (Figure S1D). We show that treatment failure is associated with both quantitative accumulation and distinct regulatory reprogramming of NCMs. Notably, cells from non‐responders exhibit a transcriptional signature consistent with terminal differentiation and cellular senescence (Figures 2G,H, S2D,E). Intercellular communication analysis showed that NCMs in non‐responders receive more incoming signals but emit fewer outgoing signals, acting as signal sinks rather than active communicators (Figures 3C, S3B,C). This pattern resembles exhausted or senescent cells that accumulate inhibitory inputs but fail to respond effectively [36]. Similar monocyte dysregulation has been reported in severe TB, where CD14^+^ CD16^+^ monocytes correlate with T‐cell inhibition, suggesting immune paralysis [37].

The revised sample‐level panels make individual variation visible. The major‐cell composition display reports a large standardized difference for total monocytes (Cohen's d = 1.04), but this is a descriptive estimate based on four patients per group. The distinct non‐classical‐monocyte sample panel is shown separately; we therefore do not label the total‐monocyte effect size as NCM‐specific.

Our data suggest an association between Treg‐associated GALECTIN signalling and exhaustion‐associated transcriptional features in cytotoxic lymphocytes [38]. The LGALS9 correlations with HAVCR2 and TIGIT are group‐stratified cell‐level co‐expression analyses (Figure S7) and do not prove a Treg‐to‐effector causal pathway [39, 40]. The NCM‐Treg‐cytotoxic lymphocyte axis is therefore a proposed network model that requires independent protein‐level and functional testing.

These observations nominate NCM composition and IL16/TNFSF10‐related transcripts as candidate biomarkers for further study [41]. They should not be used as predictive biomarkers or host‐directed therapeutic targets without prospective validation, mechanistic experiments and evaluation of clinical feasibility and safety.

GSE40553 provides longitudinal treatment‐time context in which deconvolution‐estimated non‐classical‐monocyte proportions decline after treatment initiation. It does provide an SCC‐outcome analysis in the present study and directly validate the single‐cell functional states. We have marked the text, result heading, Figure 6 limitations to make this boundary explicit.

Together, the data support a proposed transcriptomic network in which altered NCM‐associated IL16/TNFSF10 signals may coexist with Treg‐associated GALECTIN signalling and exhaustion‐associated features in cytotoxic lymphocytes. Observational, cross‐sectional data cannot determine whether these changes are causes or consequences of persistent infection or systemic inflammation. Functional perturbation, temporal patient‐level validation and protein measurements are required before attributing failed sputum culture conversion to these pathways.

Our findings are based on transcriptional profiles, inferred intercellular communication, cell‐level co‐expression, trajectory inference and exploratory in silico perturbation. We did not measure IL16, TNFSF10 or LGALS9 protein and did not perform experimental gene perturbation. Trajectory methods may be sensitive to sampling density, patient heterogeneity and modelling assumptions; the terminal‐differentiation/senescence‐like interpretation should therefore be considered provisional.

**FIGURE 7:**
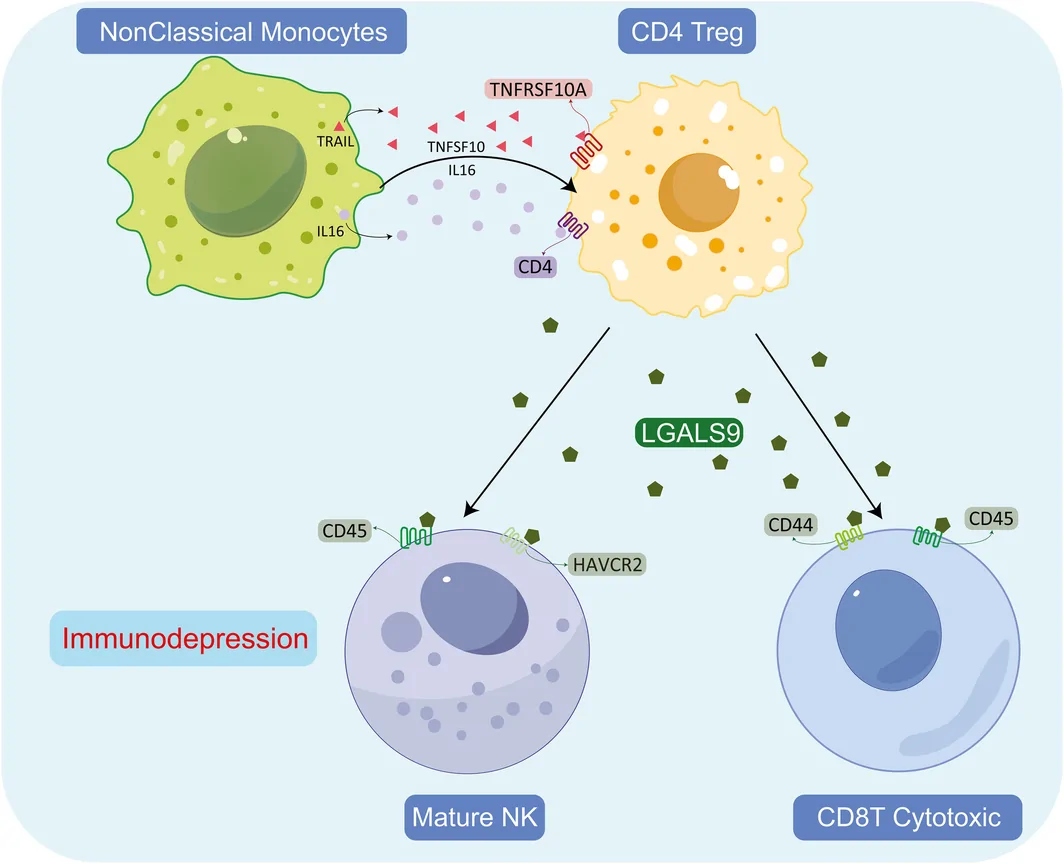
CellChat‐based inference identified putative IL16/TNFSF10 signalling from non‐classical monocytes and putative LGALS9‐associated signalling from Tregs to cytotoxic lymphocyte subsets. These computational observations support a candidate intercellular communication model but do not establish direct cellular interactions or a causal sequence.

We acknowledge that the modest sample size (*n* = 8; four patients per group) limits precision and generalizability. Patient‐level NCM composition and IL16/TNFSF10 expression display document inter‐individual variation; however, sample‐level LGALS9 expression data were not available for an independent patient‐level test. With four patients per group, Cohen's d is descriptive and no confidence interval was available from the supplied analysis. Larger prospective cohorts with individual SCC outcomes, protein measurements and functional assays are required to assess reproducibility, clinical utility and mechanism (Figure 7).

## Author Contributions

**Yiqun Xiong:** data curation, writing – original draft. **Yahong Qu:** writing – original draft, writing – review and editing. **Ying Wang:** writing – review and editing, formal analysis. **Dongliang Zhang:** writing – review and editing, formal analysis. **Hao Shen:** conceptualization, data curation, writing – review and editing. **Weixin Wang:** writing – review and editing, conceptualization, data curation. **Junshun Gao:** conceptualization, writing – review and editing, data curation. **ZhenBo Wang:** conceptualization, writing – original draft, writing – review and editing. **Zhihong Shen:** supervision, writing – review and editing. **Yang Che:** writing – original draft, writing – review and editing, funding acquisition, conceptualization.

## Funding

This research was supported by Zhejiang Provincial Natural Science Foundation of China under Grant No. LTGY23H190001, Ningbo Public Welfare Science and Technology Program Project (No. 2024S040), Ningbo Top Medical and Health Research Program (No. 2023020713). The funder had no role in study design, data collection, analysis, interpretation of data and writing the manuscript.

## Ethics Statement

This study was approved by the Ethics Committee of the Ningbo Municipal Center for Disease Control and Prevention.

## Consent

All eligible participants who agreed to participate in the program and signed an informed consent form were required to complete a questionnaire and provide specimens for subsequent studies.

## Conflicts of Interest

The authors declare no conflicts of interest.

## Supporting information


**Figure S1:** Supplementary analyses supporting the cellular and molecular differences between TB treatment responders and non‐responders. (A) Clinical characteristics of patients in Control and Case groups, including demographic and treatment‐related variables. (B) Expression of canonical marker genes used to annotate major immune cell types. (C) Relative proportions of immune cell subsets across individual samples. (D) Expression patterns of selected genes in monocytes across groups. (E) Gene set scoring of immune‐related pathways comparing Control and Case groups.

**Figure S2:** Supplementary analyses of the myeloid compartment and non‐classical monocyte differentiation. (A) Dot plot visualization of myeloid subclusters with corresponding annotations. (B) Box plots showing relative frequencies of each myeloid subset across samples. (C) Violin plots showing TB progression risk scores across myeloid subsets in each sample. (D) Monocle3 pseudotime trajectory plots showing differentiation path among myeloid subsets. (E) CytoTRACE analysis indicating differentiation states of myeloid subsets. (F) Dot plot of transcription factor activity inferred by SCENIC analysis in non‐classical monocytes.

**Figure S3:** Intercellular communication analysis comparing Case and Control groups. (A) Bar plot showing total interaction counts and overall communication strength across all cell types in Case and Control groups. (B) Heatmap comparing the number and strength of different interactions between Case and Control groups. (C) Heatmap illustrating overall signalling pathway strengths in Case versus Control groups.

**Figure S4:** Differential expression of immunoregulatory pathways in non‐classical monocytes across patient subgroups. (A–C) Gene expression levels of IL16, TNFSF10 and TNFRSF10A across the three patient subgroups identified by trajectory‐based clustering. (D) Expression of LGALS9 across the three subgroups. Statistical comparisons were performed using the Wilcoxon rank‐sum test.

**Figure S5:** Expression of exhaustion‐associated markers across T cell subsets in Responder and Non‐responder groups.(A‐B) UMAP plots showing HAVCR2 (TIM‐3) expression intensity in Responder (A) and Non‐responder (B) groups. Colour gradient indicates normalized expression levels. (C) Bubble plot displaying the mean expression of HAVCR2 across T cell subsets. Bubble size represents the proportion of cells expressing HAVCR2 within each subset, and colour intensity reflects the mean expression level. (D‐E) UMAP plots showing TIGIT expression intensity in Responder (D) and Non‐responder (E) groups. (F) Bubble plot displaying the mean expression of TIGIT across T cell subsets. Bubble size represents the proportion of cells expressing TIGIT within each subset, and colour intensity reflects the mean expression level.

**Figure S6:** Patient‐level composition, expression and effect‐size displays. (A) NCM composition for each non‐responder and responder sample (*n* = 4 per group). (B) Patient‐level IL16 and TNFSF10 expression summaries. (C) Cohen's d estimates for major cell‐type proportions; the total‐monocyte comparison was d = 1.04. Each point in A and B represents one biological replicate.

**Figure S7:** Within‐group cellular correlations between LGALS9 and exhaustion‐associated genes. (A) LGALS9‐HAVCR2 correlation. (B) LGALS9‐TIGIT correlation. Correlation coefficients and two‐sided *p* values are shown for non‐responder and responder cells. These analyses are cell‐level co‐expression analyses, not patient‐level association tests.

**Figure S8:** KEGG enrichment of Mature NK subcluster‐specific gene sets. (A) Mature NK c1. (B) Mature NK c2. Bars show ‐log10 adjusted *p* values; values in parentheses indicate overlapping genes divided by pathway genes. Results characterize subcluster gene sets and are not treatment‐group comparisons.

**Figure S9:** Exploratory scTenifoldKnk analysis of IL16 and TNFSF10. (A) Representative FDR‐significant perturbation‐associated genes after IL16 virtual knockout. (B) Representative FDR‐significant perturbation‐associated genes after TNFSF10 virtual knockout. Values are ‐log10 adjusted *p* values and exclude the targeted gene. (C) Overlap between perturbation‐associated gene sets and GO biological‐process enrichment of mapped shared genes. This analysis is hypothesis‐generating and does not substitute for experimental perturbation or protein validation.

**Table S1:** Clinical characteristics of study participants included in the single‐cell RNA sequencing analysis. Information includes group allocation (case vs. control), gender, age, anthropometric measurements (weight, height), tuberculosis treatment history, lifestyle factors (smoking, alcohol use), comorbidities, baseline medication regimen, baseline drug resistance profile, baseline microbiology results, 2‐month sputum culture conversion status and overall treatment outcome.

**Table S2:** Single‐cell RNA sequencing metadata and differential gene expression results. Sheet1_MetaData: Per‐cell quality control metrics, including total and mitochondrial UMI counts, ribosomal counts, number of detected genes, doublet scores, clustering assignments and annotated cell type and sub‐cell type identities. Sheet2_CelltypeMarker: Marker genes for each annotated cell type, with associated scores, log fold‐changes, *p*‐values, adjusted *p*‐values and detection rates in group vs. reference. Sheet3_GroupMarker: Differentially expressed genes between case and control groups, with corresponding scores, log fold‐changes, *p*‐values, adjusted *p*‐values and detection rates.

**Table S3:** Gene sets used in the study for tuberculosis risk signature and immune response analyses. This table lists curated biological pathways and functional gene sets, including Risk signature for tuberculosis progression, Activation of immune response, Activation of innate immune response, Adaptive immune response, Antifungal humoral response, Antifungal innate immune response, Antiviral innate immune response and Antigen processing and presentation.

**Table S4:** Curated gene modules used for functional scoring of T‐cell states. Each column contains genes assigned to inhibitory, cytotoxicity, co‐stimulatory, pre‐dysfunctional, resident or naive T‐cell programs.

**Table S5:** scTenifoldKnk virtual knockout results for IL16 and TNFSF10. The IL16_significant and TNFSF10_significant worksheets list FDR‐significant genes with altered network regulation after virtual knockout, including perturbation distance, Z score, fold change (FC), nominal *p* value, and adjusted *p* value. The Shared_genes worksheet lists the 90 genes significant in both analyses.

## Data Availability

The datasets generated during the current study will be available in the National Genomics Data Center (NGDC) repository (https://ngdc.cncb.ac.cn/) under accession number PRJCA044651 upon completion of submission. Reasonable requests for data during the submission process should be directed to the corresponding author. Bulk RNA‐seq raw and normalized microarray data have been deposited with the GEO (GSE40553).
