# Supplementary material for: Single‐Cell Immune Profiling Suggests Non‐Classical Monocyte Is Associated Immunodepression in Tuberculosis Treatment Non‐Responders
Source: J Cell Mol Med. 2026 Aug 31;30(17):e71345. doi: 10.1111/jcmm.71345 (PMC13530280; doi:10.1111/jcmm.71345)
Supplement: Supplementary file 1 [file JCMM-30-e71345-s002.pdf]

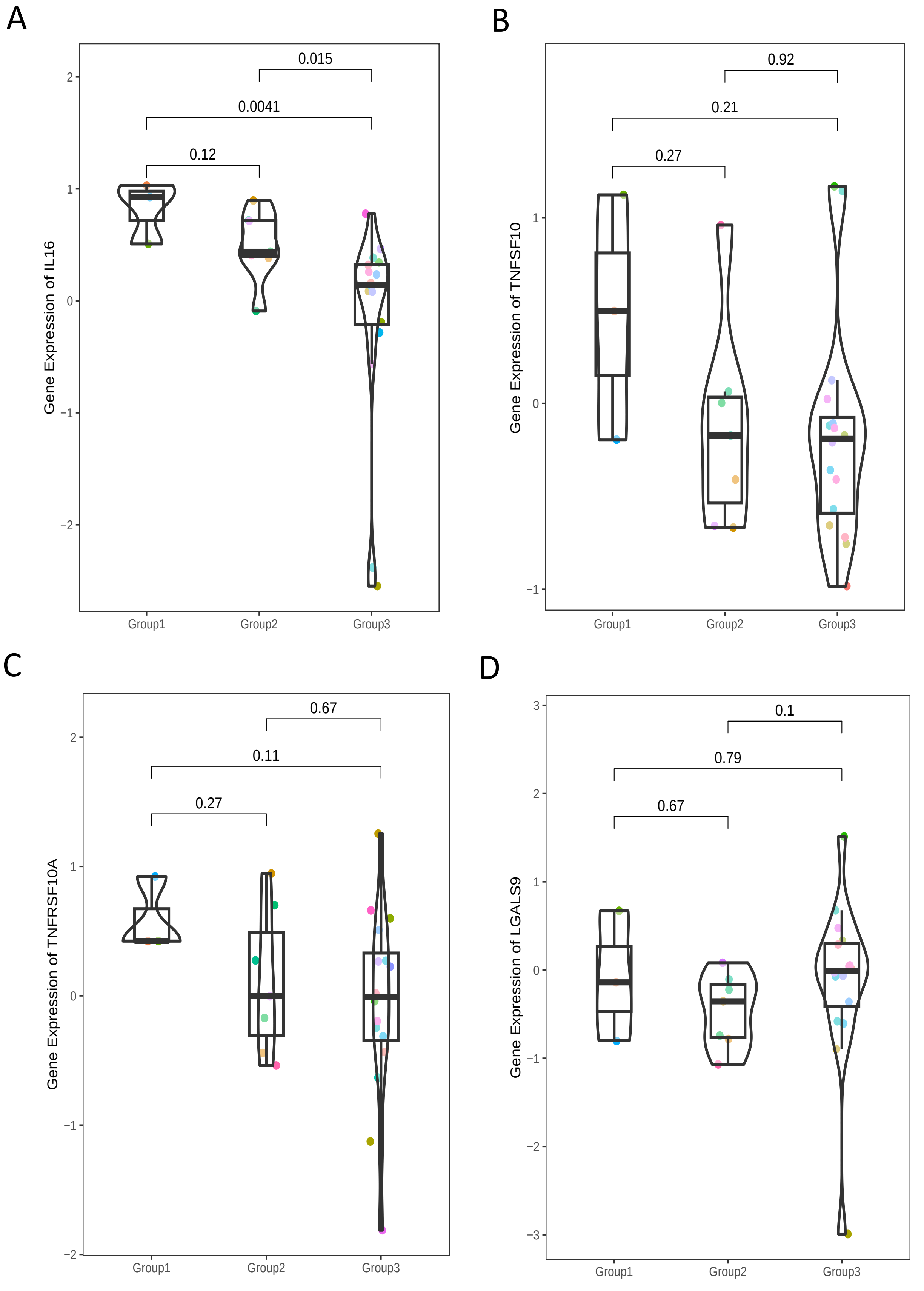
A
B
C
D
Gene Expression of IL16
Gene Expression of TNFSF10
Gene Expression of TNFRSF10A
Gene Expression of LGALS9
0.015
0.0041
0.12
0.92
0.21
0.27
0.67
0.11
0.27
0.1
0.79
0.67
Group1
Group2
Group3
