# Supplementary material for: Single‐Cell Immune Profiling Suggests Non‐Classical Monocyte Is Associated Immunodepression in Tuberculosis Treatment Non‐Responders
Source: J Cell Mol Med. 2026 Aug 31;30(17):e71345. doi: 10.1111/jcmm.71345 (PMC13530280; doi:10.1111/jcmm.71345)
Supplement: Supplementary file 2 [file JCMM-30-e71345-s003.pdf]

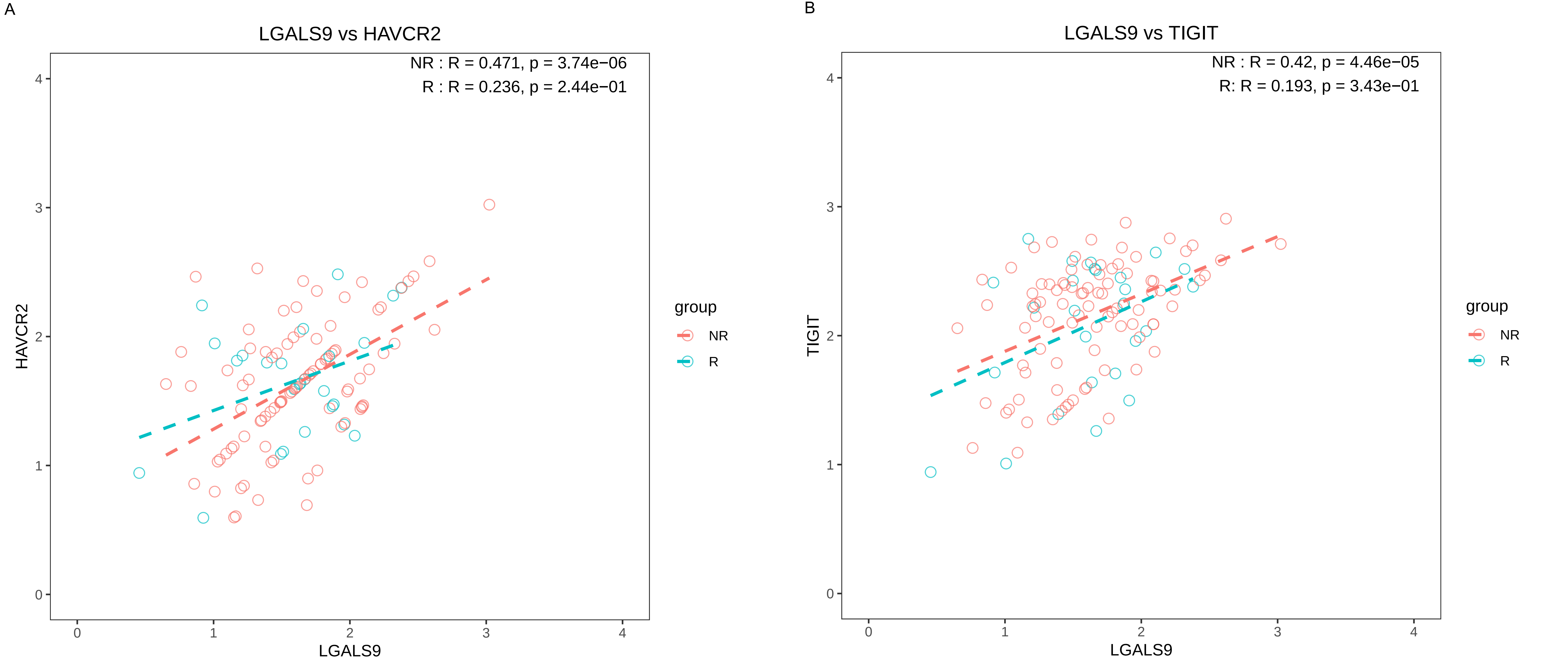

A
LGALS9 vs HAVCR2
NR : R = 0.471, p = 3.74e−06
R : R = 0.236, p = 2.44e−01
HAVCR2
LGALS9
0
1
2
3
4
group
NR
R
B
LGALS9 vs TIGIT
NR : R = 0.42, p = 4.46e−05
R: R = 0.193, p = 3.43e−01
TIGIT
LGALS9
group
NR
R
