# Supplementary material for: Single‐Cell Immune Profiling Suggests Non‐Classical Monocyte Is Associated Immunodepression in Tuberculosis Treatment Non‐Responders
Source: J Cell Mol Med. 2026 Aug 31;30(17):e71345. doi: 10.1111/jcmm.71345 (PMC13530280; doi:10.1111/jcmm.71345)
Supplement: Supplementary file 3 [file JCMM-30-e71345-s004.pdf]

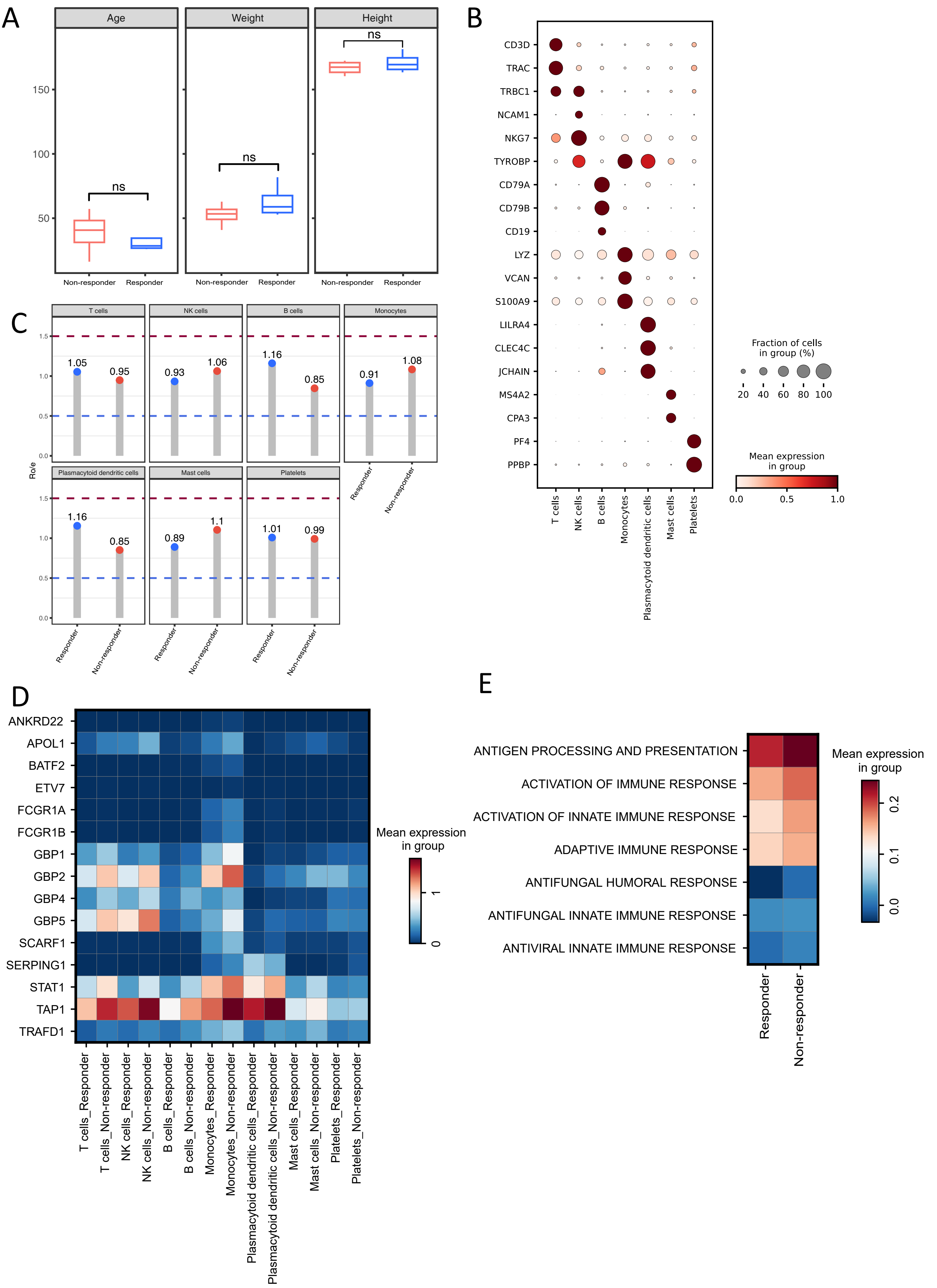
A
Age
Weight
Height
ns
150
100
50
Non-responder
Responder
B
CD3D
TRAC
TRBC1
NCAM1
NKG7
TYROBP
CD79A
CD79B
CD19
LYZ
VCAN
S100A9
LILRA4
CLEC4C
JCHAIN
MS4A2
CPA3
PF4
PPBP
T cells
NK cells
B cells
Monocytes
Plasmacytoid dendritic cells
Mast cells
Platelets
Fraction of cells in group (%)
20 40 60 80 100
Mean expression in group
0.0 0.5 1.0
C
Ro/e
1.05 0.95
0.93 1.06
1.16 0.85
0.91 1.08
1.16 0.85
0.89 1.1
1.01 0.99
D
ANKRD22
APOL1
BATF2
ETV7
FCGR1A
FCGR1B
GBP1
GBP2
GBP4
GBP5
SCARF1
SERPING1
STAT1
TAP1
TRAFD1
T cells_Responder
T cells_Non-responder
NK cells_Responder
NK cells_Non-responder
B cells_Responder
B cells_Non-responder
Monocytes_Responder
Monocytes_Non-responder
Plasmacytoid dendritic cells_Responder
Plasmacytoid dendritic cells_Non-responder
Mast cells_Responder
Mast cells_Non-responder
Platelets_Responder
Platelets_Non-responder
Mean expression in group
E
ANTIGEN PROCESSING AND PRESENTATION
ACTIVATION OF IMMUNE RESPONSE
ACTIVATION OF INNATE IMMUNE RESPONSE
ADAPTIVE IMMUNE RESPONSE
ANTIFUNGAL HUMORAL RESPONSE
ANTIFUNGAL INNATE IMMUNE RESPONSE
ANTIVIRAL INNATE IMMUNE RESPONSE
Responder
Non-responder
Mean expression in group
