# Supplementary material for: Single‐Cell Immune Profiling Suggests Non‐Classical Monocyte Is Associated Immunodepression in Tuberculosis Treatment Non‐Responders
Source: J Cell Mol Med. 2026 Aug 31;30(17):e71345. doi: 10.1111/jcmm.71345 (PMC13530280; doi:10.1111/jcmm.71345)
Supplement: Supplementary file 4 [file JCMM-30-e71345-s005.pdf]

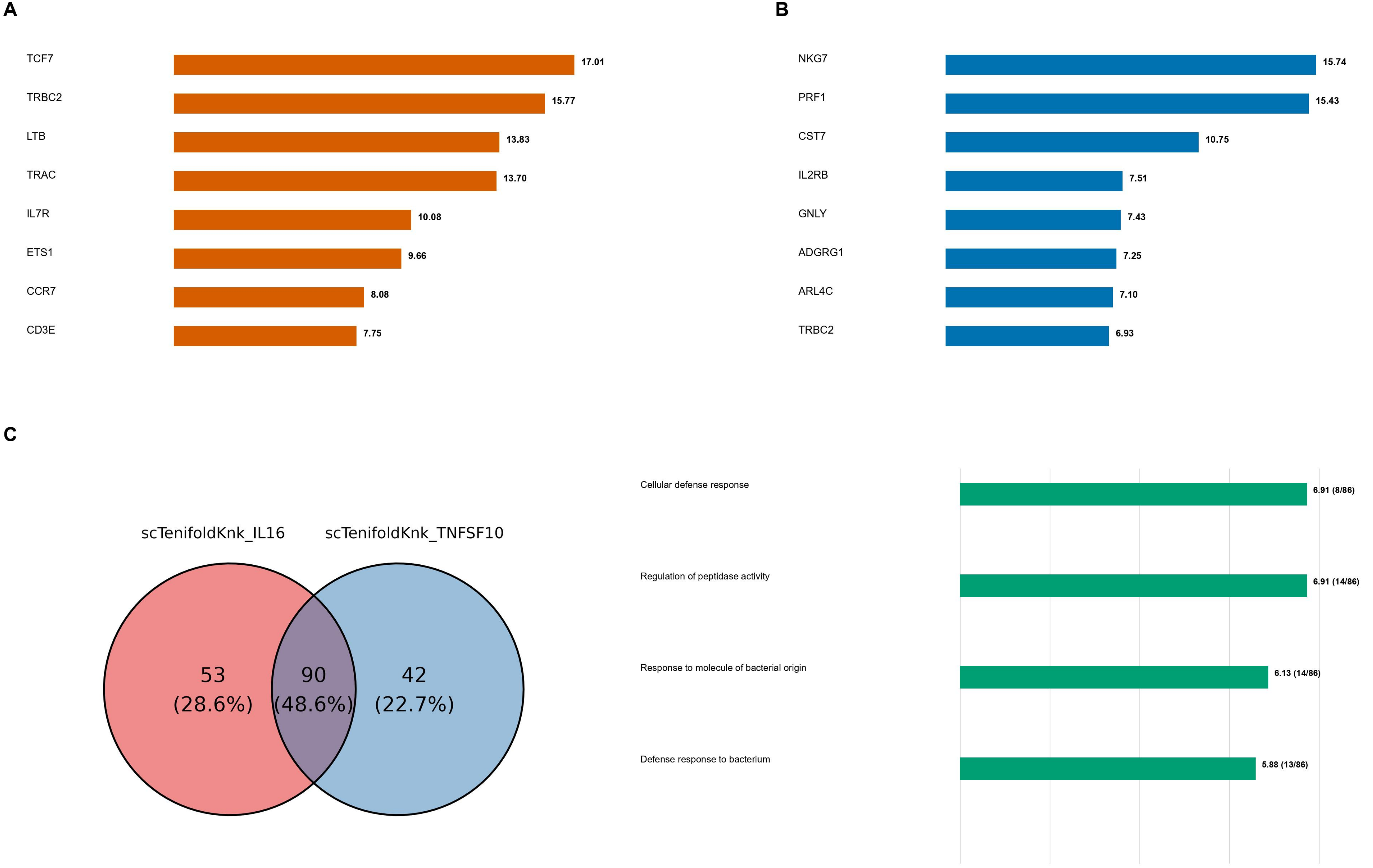
A
TCF7
17.01
TRBC2
15.77
LTB
13.83
TRAC
13.70
IL7R
10.08
ETS1
9.66
CCR7
8.08
CD3E
7.75
B
NKG7
15.74
PRF1
15.43
CST7
10.75
IL2RB
7.51
GNLY
7.43
ADGRG1
7.25
ARL4C
7.10
TRBC2
6.93
C
scTenifoldKnk_IL16
scTenifoldKnk_TNFSF10
53
(28.6%)
90
(48.6%)
42
(22.7%)
Cellular defense response
6.91 (8/86)
Regulation of peptidase activity
6.91 (14/86)
Response to molecule of bacterial origin
6.13 (14/86)
Defense response to bacterium
5.88 (13/86)
