# Supplementary material for: Single‐Cell Immune Profiling Suggests Non‐Classical Monocyte Is Associated Immunodepression in Tuberculosis Treatment Non‐Responders
Source: J Cell Mol Med. 2026 Aug 31;30(17):e71345. doi: 10.1111/jcmm.71345 (PMC13530280; doi:10.1111/jcmm.71345)
Supplement: Supplementary file 5 [file JCMM-30-e71345-s007.pdf]

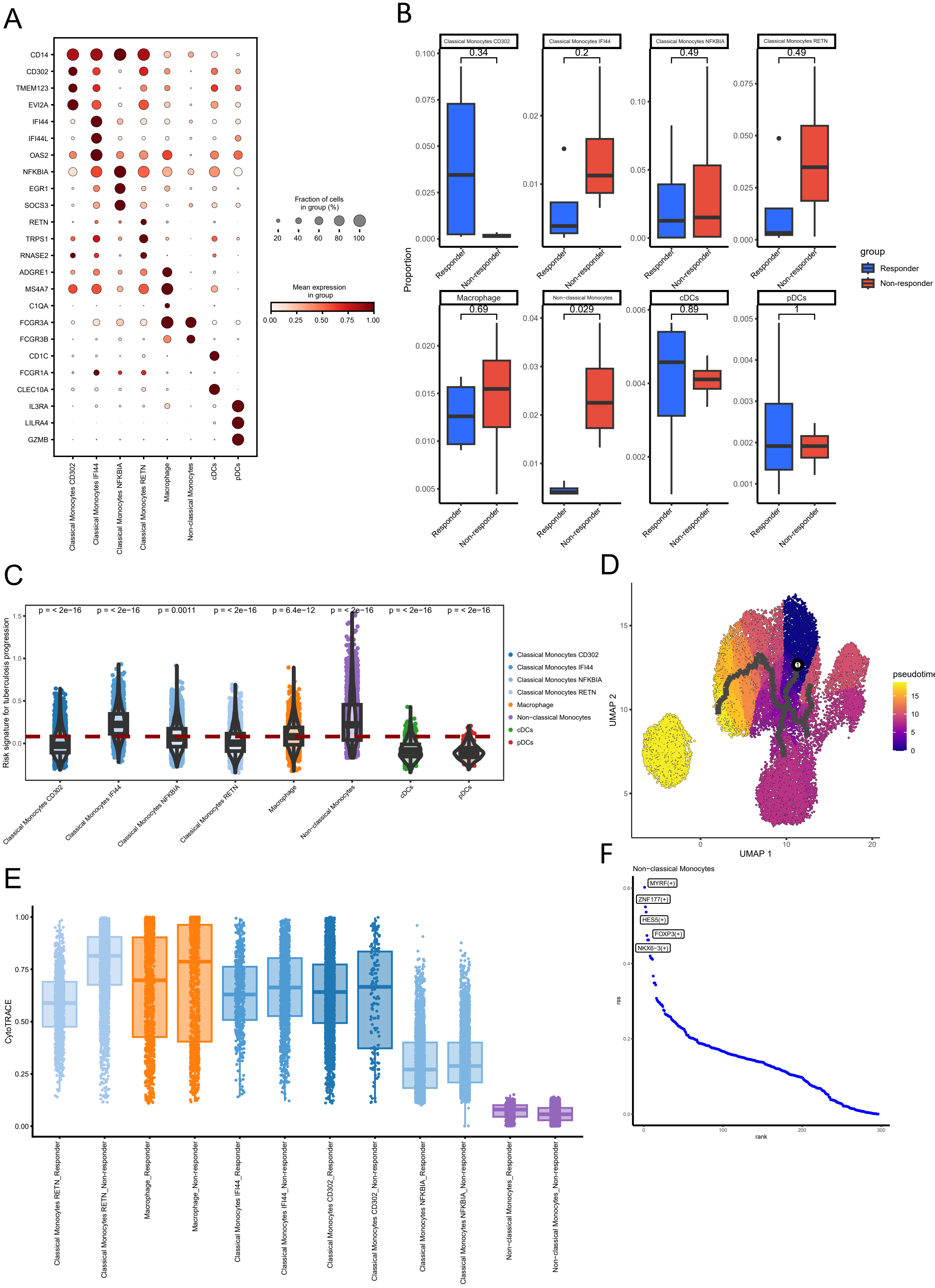

A
CD14
CD302
TMEM123
EVI2A
IFI44
IFI44L
OAS2
NFKBIA
EGR1
SOCS3
RETN
TRPS1
RNASE2
ADGRE1
MS4A7
C1QA
FCGR3A
FCGR3B
CD1C
FCGR1A
CLEC10A
IL3RA
LILRA4
GZMB
Classical Monocytes CD302
Classical Monocytes IFI44
Classical Monocytes NFKBIA
Classical Monocytes RETN
Macrophage
Non-classical Monocytes
cDCs
pDCs
Fraction of cells in group (%)
20 40 60 80 100
Mean expression in group
0.00 0.25 0.50 0.75 1.00
B
Proportion
Classical Monocytes CD302
0.34
Classical Monocytes IFI44
0.2
Classical Monocytes NFKBIA
0.49
Classical Monocytes RETN
0.49
Macrophage
0.69
Non-classical Monocytes
0.029
cDCs
0.89
pDCs
1
Responder
Non-responder
group
Responder
Non-responder
C
Risk signature for tuberculosis progression
p = < 2e−16
p = < 2e−16
p = 0.0011
p = < 2e−16
p = 6.4e−12
p = < 2e−16
p = < 2e−16
p = < 2e−16
Classical Monocytes CD302
Classical Monocytes IFI44
Classical Monocytes NFKBIA
Classical Monocytes RETN
Macrophage
Non-classical Monocytes
cDCs
pDCs
D
UMAP 1
UMAP 2
pseudotime
0 5 10 15
E
CytoTRACE
Classical Monocytes RETN_Responder
Classical Monocytes RETN_Non-responder
Macrophage_Responder
Macrophage_Non-responder
Classical Monocytes IFI44_Responder
Classical Monocytes IFI44_Non-responder
Classical Monocytes CD302_Responder
Classical Monocytes CD302_Non-responder
Classical Monocytes NFKBIA_Responder
Classical Monocytes NFKBIA_Non-responder
Non-classical Monocytes_Responder
Non-classical Monocytes_Non-responder
F
Non-classical Monocytes
MYRF(+)
ZNF177(+)
HES5(+)
FOXP3(+)
NKX6-3(+)
rss
rank
