# Supplementary material for: Single‐Cell Immune Profiling Suggests Non‐Classical Monocyte Is Associated Immunodepression in Tuberculosis Treatment Non‐Responders
Source: J Cell Mol Med. 2026 Aug 31;30(17):e71345. doi: 10.1111/jcmm.71345 (PMC13530280; doi:10.1111/jcmm.71345)
Supplement: Supplementary file 6 [file JCMM-30-e71345-s008.pdf]

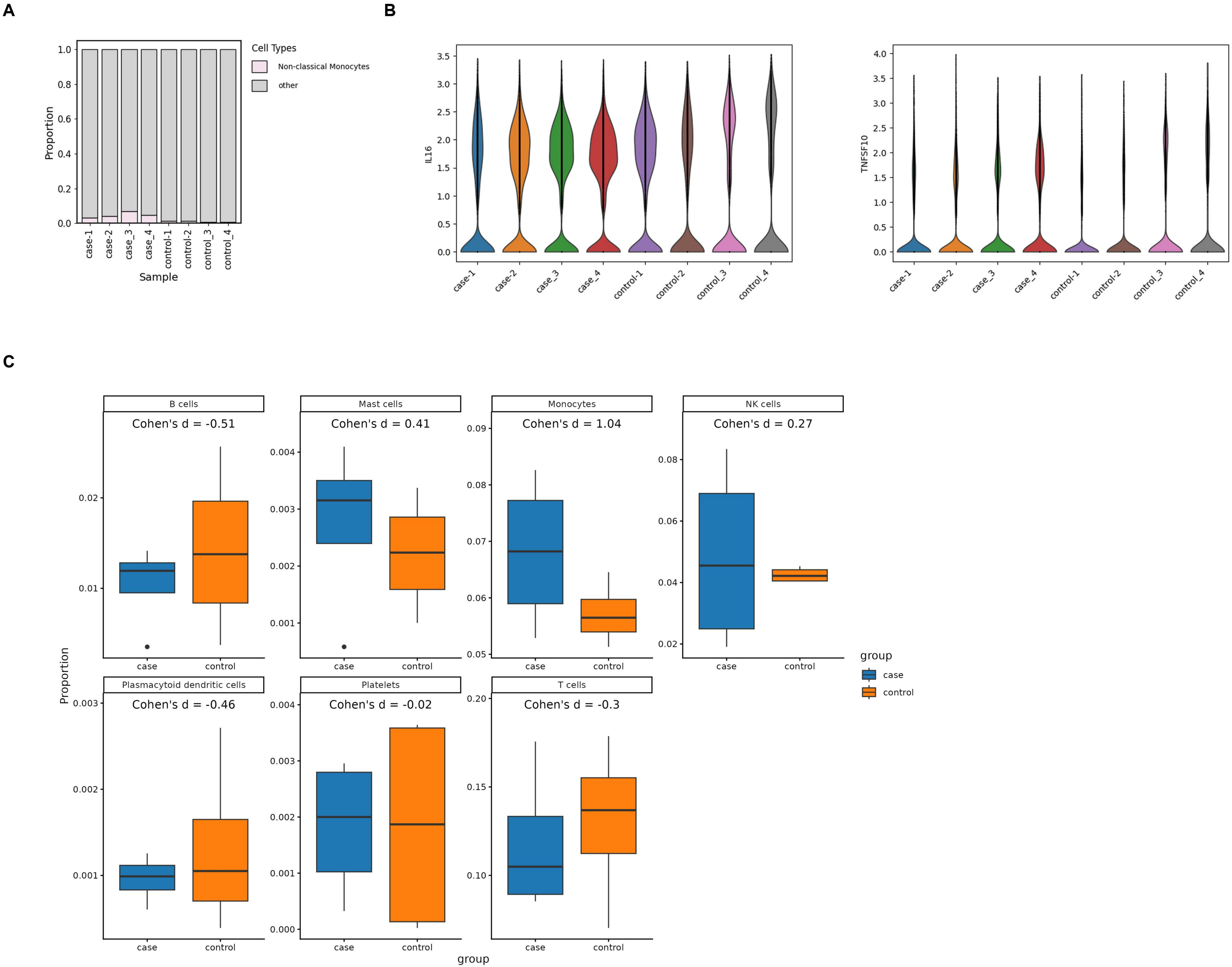
A
B
C
Cell Types
Non-classical Monocytes
other
Proportion
Sample
case-1
case-2
case_3
case_4
control-1
control-2
control_3
control_4
IL16
TNFSF10
B cells
Cohen's d = -0.51
Mast cells
Cohen's d = 0.41
Monocytes
Cohen's d = 1.04
NK cells
Cohen's d = 0.27
Plasmacytoid dendritic cells
Cohen's d = -0.46
Platelets
Cohen's d = -0.02
T cells
Cohen's d = -0.3
case
control
group
