# Supplementary material for: Single‐Cell Immune Profiling Suggests Non‐Classical Monocyte Is Associated Immunodepression in Tuberculosis Treatment Non‐Responders
Source: J Cell Mol Med. 2026 Aug 31;30(17):e71345. doi: 10.1111/jcmm.71345 (PMC13530280; doi:10.1111/jcmm.71345)
Supplement: Supplementary file 7 [file JCMM-30-e71345-s009.pdf]

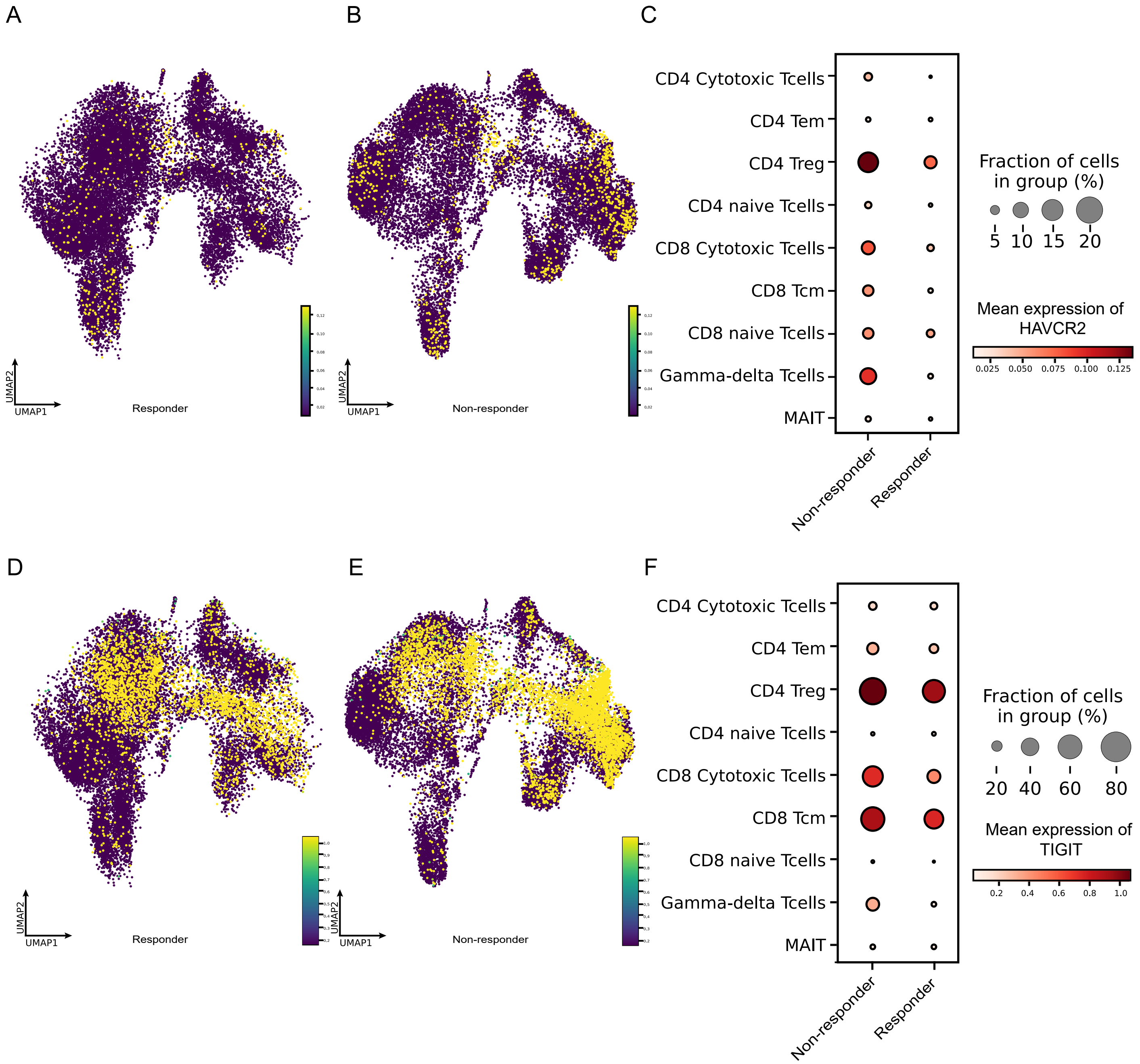
A
B
C
UMAP2
UMAP1
Responder
Non-responder
CD4 Cytotoxic Tcells
CD4 Tem
CD4 Treg
CD4 naive Tcells
CD8 Cytotoxic Tcells
CD8 Tcm
CD8 naive Tcells
Gamma-delta Tcells
MAIT
Non-responder
Responder
Fraction of cells in group (%)
5 10 15 20
Mean expression of HAVCR2
0.025 0.050 0.075 0.100 0.125
D
E
F
Fraction of cells in group (%)
20 40 60 80
Mean expression of TIGIT
0.2 0.4 0.6 0.8 1.0
