# Supplementary material for: Single‐Cell Immune Profiling Suggests Non‐Classical Monocyte Is Associated Immunodepression in Tuberculosis Treatment Non‐Responders
Source: J Cell Mol Med. 2026 Aug 31;30(17):e71345. doi: 10.1111/jcmm.71345 (PMC13530280; doi:10.1111/jcmm.71345)
Supplement: Supplementary file 8 [file JCMM-30-e71345-s010.pdf]

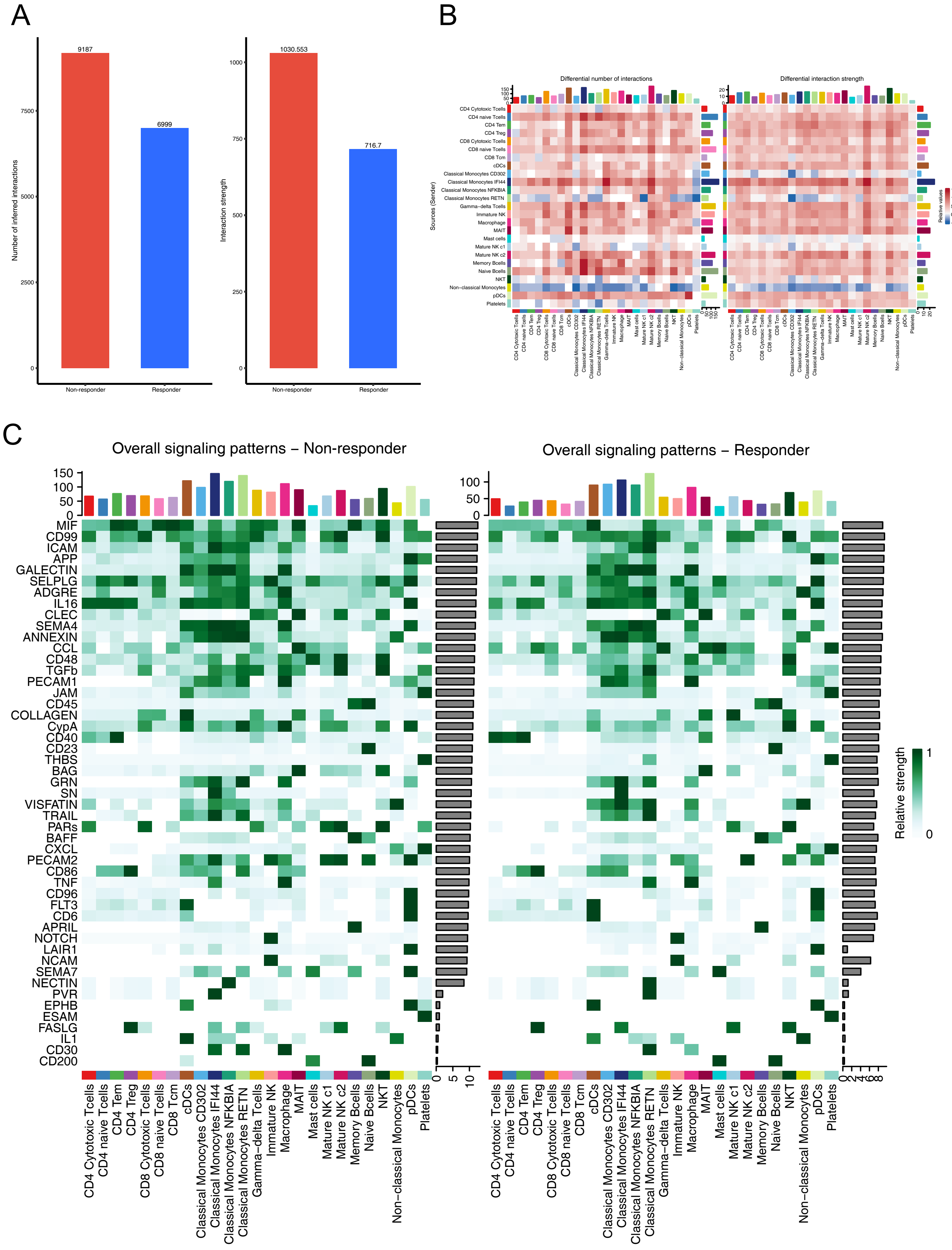

A
9187
6999
Number of inferred interactions
Non-responder
Responder
1030.553
716.7
Interaction strength
B
Differential number of interactions
Differential interaction strength
Sources (Sender)
Relative values
C
Overall signaling patterns − Non-responder
Overall signaling patterns − Responder
Relative strength
MIF
CD99
ICAM
APP
GALECTIN
SELPLG
ADGRE
IL16
CLEC
SEMA4
ANNEXIN
CCL
CD48
TGFb
PECAM1
JAM
CD45
COLLAGEN
CypA
CD40
CD23
THBS
BAG
GRN
SN
VISFATIN
TRAIL
PARs
BAFF
CXCL
PECAM2
CD86
TNF
CD96
FLT3
CD6
APRIL
NOTCH
LAIR1
NCAM
SEMA7
NECTIN
PVR
EPHB
ESAM
FASLG
IL1
CD30
CD200
CD4 Cytotoxic Tcells
CD4 naive Tcells
CD4 Tem
CD4 Treg
CD8 Cytotoxic Tcells
CD8 naive Tcells
CD8 Tcm
cDCs
Classical Monocytes CD302
Classical Monocytes IFI44
Classical Monocytes NFKBIA
Classical Monocytes RETN
Gamma−delta Tcells
Immature NK
Macrophage
MAIT
Mast cells
Mature NK c1
Mature NK c2
Memory Bcells
Naive Bcells
NKT
Non−classical Monocytes
pDCs
Platelets
