# Supplementary material for: Single‐Cell Immune Profiling Suggests Non‐Classical Monocyte Is Associated Immunodepression in Tuberculosis Treatment Non‐Responders
Source: J Cell Mol Med. 2026 Aug 31;30(17):e71345. doi: 10.1111/jcmm.71345 (PMC13530280; doi:10.1111/jcmm.71345)
Supplement: Supplementary file 9 [file JCMM-30-e71345-s012.pdf]

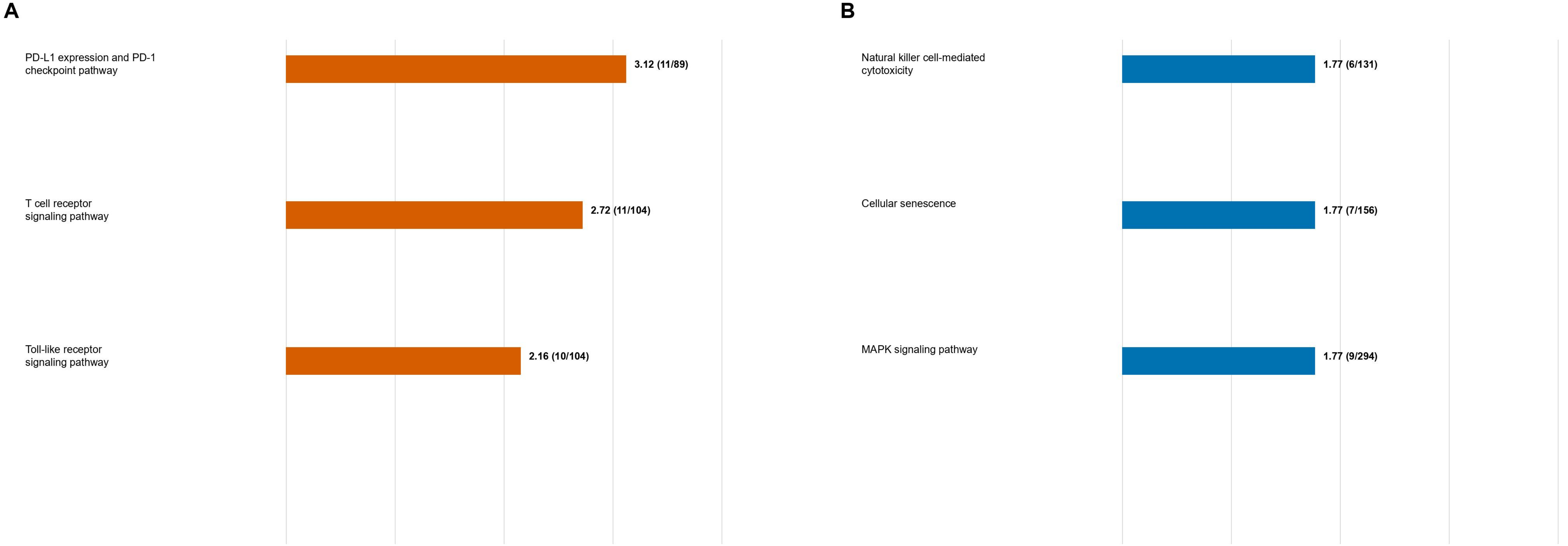

A
PD-L1 expression and PD-1 checkpoint pathway
3.12 (11/89)
T cell receptor signaling pathway
2.72 (11/104)
Toll-like receptor signaling pathway
2.16 (10/104)
B
Natural killer cell-mediated cytotoxicity
1.77 (6/131)
Cellular senescence
1.77 (7/156)
MAPK signaling pathway
1.77 (9/294)
